# Targeting Hodgkin and Reed–Sternberg Cells with an Inhibitor of Heat-Shock Protein 90: Molecular Pathways of Response and Potential Mechanisms of Resistance

**DOI:** 10.3390/ijms19030836

**Published:** 2018-03-13

**Authors:** Priscilla Segges, Stephany Corrêa, Bárbara Du Rocher, Gabriela Vera-Lozada, Flavia Krsticevic, Debora Arce, Cinthya Sternberg, Eliana Abdelhay, Rocio Hassan

**Affiliations:** 1 Oncovirology Laboratory, Bone Marrow Transplantation Center (CEMO), Instituto Nacional de Câncer (INCA), Rio de Janeiro 20230-130, Brazil; gabrielavl810@gmail.com (G.V.-L.); fkrsticevic@gmail.com (F.K.); chassan@inca.gov.br (R.H.); 2 Laboratório Célula-Tronco, CEMO, Instituto Nacional de Câncer (INCA), Rio de Janeiro 20230-130, Brazil; stephy_correa@yahoo.com.br (S.C.); barbaradurocher@ymail.com (B.D.R.); eabdelhay@inca.gov.br (E.A.); 3 Laboratório de Pesquisa sobre o Timo, FIOCRUZ, Rio de Janeiro 21040-900, Brazil; 4 CIFASIS—Centro Internacional Franco Argentino de Ciencias de la Información y de Sistemas, Rosario 4237248, Argentina; debora.arce@gmail.com; 5 IICAR-CONICET Instituto de Ciencias Agrarias de Rosario, Rosario 4237248, Argentina; 6 Laboratório de Pesquisa Translacional, Instituto Nacional de Câncer (INCA), Rio de Janeiro 20230-130, Brazil; diretoriaexecutiva@sboc.org.br

**Keywords:** celastrol, Hodgkin lymphoma, heat-shock protein 90 (HSP90) inhibition, label-free proteomics, MAPK/ERK pathway, HSP27

## Abstract

Classical Hodgkin lymphoma (cHL) cells overexpress heat-shock protein 90 (HSP90), an important intracellular signaling hub regulating cell survival, which is emerging as a promising therapeutic target. Here, we report the antitumor effect of celastrol, an anti-inflammatory compound and a recognized HSP90 inhibitor, in Hodgkin and Reed–Sternberg cell lines. Two disparate responses were recorded. In KM-H2 cells, celastrol inhibited cell proliferation, induced G0/G1 arrest, and triggered apoptosis through the activation of caspase-3/7. Conversely, L428 cells exhibited resistance to the compound. A proteomic screening identified a total of 262 differentially expressed proteins in sensitive KM-H2 cells and revealed that celastrol’s toxicity involved the suppression of the MAPK/ERK (extracellular signal regulated kinase/mitogen activated protein kinase) pathway. The apoptotic effects were preceded by a decrease in RAS (proto-oncogene protein Ras), p-ERK1/2 (phospho-extracellular signal-regulated Kinase-1/2), and c-Fos (proto-oncogene protein c-Fos) protein levels, as validated by immunoblot analysis. The L428 resistant cells exhibited a marked induction of HSP27 mRNA and protein after celastrol treatment. Our results provide the first evidence that celastrol has antitumor effects in cHL cells through the suppression of the MAPK/ERK pathway. Resistance to celastrol has rarely been described, and our results suggest that in cHL it may be mediated by the upregulation of HSP27. The antitumor properties of celastrol against cHL and whether the disparate responses observed in vitro have clinical correlates deserve further research.

## 1. Introduction

Classical Hodgkin lymphoma (cHL) tumors cells are crippled germinal center B cells found at low numbers in tumor masses (up to 1%) amidst a vast majority of non-malignant reactive cells [1]. Collectively named as Hodgkin (uninucleated) and Reed–Sternberg (bi–multinucleated) (H-RS) cells, tumor cells exhibit a loss of the pan B cell phenotype [2] and uniformly express the CD30 antigen [3], a member of the tumor necrosis factor receptor (TNFR) family [4]. CD30 overexpression results in ligand-independent constitutive signaling that activates the NF-κB (nuclear factor NF-κB) transcription factors and the activator protein-1 (AP-1), which are critical pathways for H-RS cell survival [5,6].

With current therapeutic protocols, on average, 70% of cHL patients are cured, as the cells are sensitive to irradiation and chemotherapeutic agents [7]. However, relapsed or refractory disease has a poor prognosis, and no efficient therapeutic approaches are widely available. In addition, the current therapy is associated with toxicity and late side effects, such as secondary malignancies and cardiac toxicity [7,8], which stimulates the continuing goal of finding novel, efficient, and less toxic therapeutic molecules.

Celastrol, a triterpene derived from the Chinese medicinal plant *Triterygium wilfordii* (Thunder of God Vine) has been used for hundreds of years to treat inflammatory conditions and, in modern times, as a dietary supplement for autoimmune diseases [9]. Recently characterized as a novel inhibitor of heat-shock protein 90 (HSP90) [9,10], celastrol has attracted great attention for its potential antitumor effects [9,11,12]. Although there are some reports on the antiproliferative activity of celastrol in leukemia [13] and lymphoblastoid cells [14], no data are available concerning the effects of celastrol on cHL. Celastrol modulates the expression of pro-inflammatory cytokines and genes regulated through the NF-κB pathways and also displays its anticancer activity by inducing the degradation of HSP90 client proteins [9,10,11]. Considering that the constitutive activation of the NF-κB pathway is a key feature of H-RS cells [5,15], celastrol may have therapeutic potential for this disease. Moreover, in Hodgkin’s lymphoma cells, HSP90 has immune regulatory activity, supports the activation of NF-κB, and has recently been identified as a central hub that integrates intracellular signaling [16,17]. Thus, HSP90 inhibition emerges as a putative therapeutic target in cHL.

In this study, we aimed to investigate the ability of celastrol to sensitize and induce apoptosis in cHL-derived cell lines. We also applied a label-free Nano-LC–MSMS (Nanoscale liquid chromatography coupled to tandem mass spectrometry) approach to reveal the potential targets being modulated by the compound. We describe distinct sensitivities of H-RS cell lines to celastrol, identifying the MAPK/ERK (Mitogen-activated protein kinases; MAPKs) pathway involvement in celastrol-mediated apoptosis, and report a differential modulation of the small heat-shock protein 27 (HSP27) expression in sensitive and resistant cells. 

## 2. Results

### 2.1. Effect of Celastrol on the Viability of KM-H2 and L428 Cells

**Figure 1:**
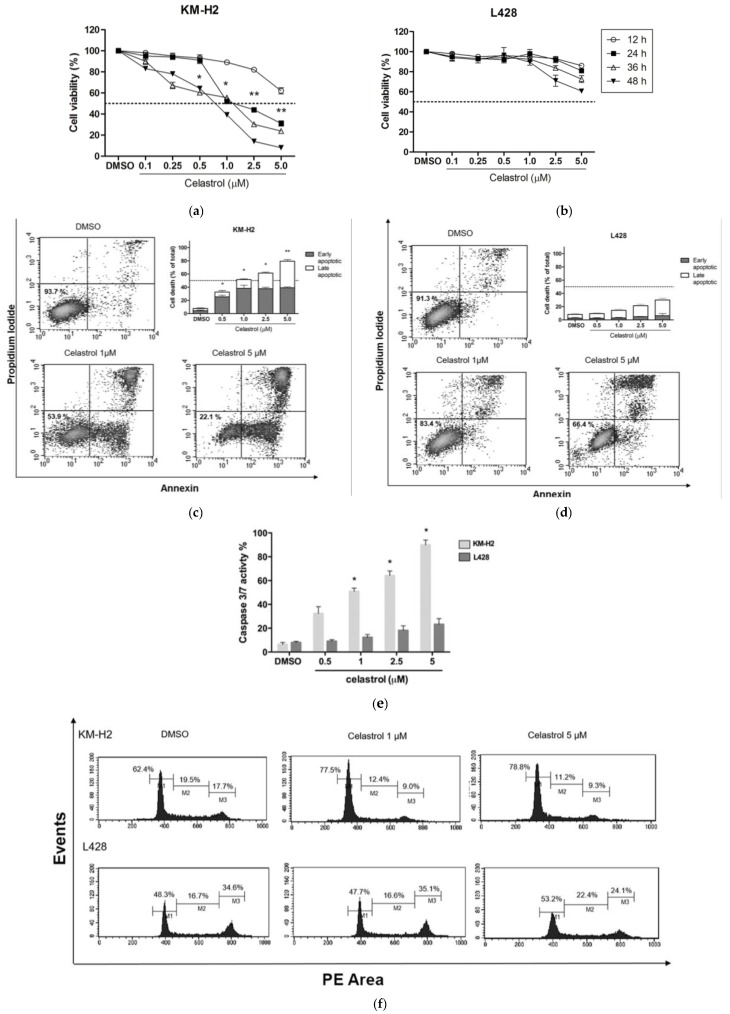
Effects of celastrol on KM-H2 and L428 cells. KM-H2 (**a**) and L428 (**b**) cell lines were treated with the indicated concentrations of celastrol or with the vehicle control (Dimethyl sulfoxide; DMSO) for 24, 48, and 72 h, and cell viability was detected by WST-1 assay (4-[3-(4-iodophenyl)-2-(4-nitrophenyl)-2*H*-5-tetrazolio]-1,3-benzene disulfonate sodium salt). Apoptosis of KM-H2 (**c**) and L428 (**d**) cell lines induced by celastrol (0.5, 1, 2.5, and 5 µM) was measured by the Annexin V assay after 24 h. Cell lines incubated with vehicle control (DMSO) were used as a control of spontaneous apoptosis. The images are representative of three independent experiments, and the means and errors of all the independent experiments are shown in the bar chart. The percentage of celastrol-induced cell death was calculated by subtracting the rates of spontaneous death determined in the control from the overall cell death rate in celastrol-treated samples for each dose point. The cells that were annexin V (+) and PI (−) were considered early-stage apoptotic cells, while annexin V (+) and PI (+) cells were considered to be in late-stage apoptosis; (**e**) profile of caspase-3/7 activation mediated by celastrol in KM-H2 and L428 cells. The percentage of caspase-3/7 activation was calculated by subtracting the values of caspase-positive samples from the negative control sample (DMSO); (**f**) changes in cell cycle induced by celastrol in KM-H2 and L428 cells. The cell lines were exposed to the indicated concentrations of celastrol and to DMSO and collected after a 24 h exposure. One experiment representative of three independent experiments is shown. The values represent the average of three independent experiments. The error bars represent ± standard error (* *p* < 0.01; ** *p* < 0.001).

Celastrol decreased cell viability in a dose-dependent manner in KM-H2. The IC50 value at 24 h treatment was 1 µM (Figure 1a). For L428 cells, celastrol did not induce significant dose-dependent reductions in cell viability, even at higher concentrations (2.5 and 5.0 µM), with a maximum reduction of 40% at 5.0 µM after 48 h of incubation (Figure 1b). This strongly suggests that celastrol has a significant cytotoxic effect in KM-H2 but not in L428 cells.

### 2.2. Celastrol Induces Apoptosis and Changes in Cell Cycle in KM-H2 but Not in L428 Cells

Exposure to celastrol for 24 h resulted in an increase of apoptosis in KM-H2 cells, including early- (Annexin V positive and PI negative) and late-stage apoptosis (Annexin V positive and PI positive). As observed in Figure 1c, apoptosis increased in a dose-dependent manner reaching 48.2% and 78.6% at concentrations of 1.0 and 5.0 µM, respectively. In contrast, celastrol treatment demonstrated only a minor, not significant, apoptotic response in L428 cells. As observed in Figure 1d, in L428 cells, apoptosis occurred mainly at a late phase with a maximum induction of 30.6% at 5 µM of celastrol.

Additionally, we assessed caspase-3 and caspase-7 activity by using a luminescent assay that measures the activity of these caspases concurrently. As indicated by the presence of cleaved substrates (Figure 1e), celastrol treatment induced caspase-3/7 activation in KM-H2 cells, whereas the treatment had no significant effects in L428 cells. Since KM-H2 cell line is caspase-3-eficient, it is likely that celastrol-induced apoptosis proceeds via caspase-7 activation.

As many HSP90 inhibitors exert their cytotoxic effects through the modulation of cell cycle progression [18], we investigated the ability of celastrol to interfere with the cell cycle. In KM-H2, treatment with celastrol for 24 h induced the accumulation of cells in G0/G1, while reducing the G2/M cell fraction (Figure 1f), suggesting that celastrol-induced apoptosis in KM-H2 is associated to alterations in the cell cycle. As expected, celastrol treatment did not result in significant changes in cell cycle distribution in L428 cells (Figure 1f).

### 2.3. Celastrol Induces Changes in the Proteome of Hodgkin’s Lymphoma Cell Lines

We next assessed the effects of celastrol in the protein expression profiles of cHL cell lines using a 2D-nanoESI-MSE (electrospray ionisation mass spectrometry) label-free protein quantitation approach. By comparing treated (TT, celastrol at 1.0 µM and incubation time of 24 h) with non-treated (in vehicle alone, NT) cells, more than 5000 peptides were identified, with a 5 ppm error for 80% of the total identified peptide from all conditions (Figure S1a). The coefficient of variation (CV) obtained for the EMRT (exact mass retention time) clusters was <4 for all conditions. The peptide match distribution showed that, on average, 5% of peptides were in source (Figure S1b) and that most of the identifications were consistent with the fragmentation in the trap CID (collision induced dissociation) cell. Moreover, a dynamic range of 3.5 log was obtained, compatible with the instrument used (Figure S1c).

Proteomic screening revealed a total of 262 and 344 differentially expressed proteins in KM-H2 and L428 cell lines, respectively, when comparing the TT and NT conditions, after the application of 2-fold change and statistic *p*-value provided by the ProteinLynx software (version 3.0.3, Waters, Milford, MA, USA; see supporting information in Tables S1–S4).

Aiming to identify modified biological processes related to each condition, bioinformatics analyses were performed with the Metacore™ software dGeneGO, Encinitas, CA, USA). All representative processes related to each subtype are described in Table S5. Most processes found in both cell lines pointed to a disturbance in metabolic pathways and proteostasis networks. Specifically, in KM-H2 cells, the first 4/5 biological processes represented well-defined groups of celastrol-induced processes that affected the general cellular organization. Altered proteins include histones, ubiquitin-protein ligase, ribosomal proteins, actin and related proteins, chromatin-binding, and membrane trafficking regulatory proteins, which revealed a markedly change in the proteome composition. Cell cycle was another process affected in the celastrol-sensitive cell line. In L428 cells, changes in differentially expressed proteins involved metabolism, protein folding and trafficking, initiation of translation, and processing of proteins in the endoplasmic reticulum.

Next, we sought to determine which pathways were significantly altered by celastrol treatment. The top 10 most relevant pathways and the differentially expressed proteins for each cell line are shown in Table 1. In KM-H2 cells, the top pathways included a network of proteins involved in mitogen-activated protein kinase (MAPK) phosphorylation, cell cycle regulation (mainly histones and MCM proteins; minichromosome maintenance protein family), signal transduction (mostly PI3K cat class IA; phosphoinositide-3-kinase, catalytic, alpha polypeptide), transcription factor (CREB-binding protein (CBP)/p300), and cytoskeleton remodeling (tubulin superfamily).

Changes in cell cycle (histones and MCM proteins) and cytoskeleton organization were also found in L428 cells. However, the cytoskeleton remodeling proteins were more numerous and variable than those observed in KM-H2 cells. As shown in Table 1, MAP kinases (mitogen-activated protein kinase) and other proteins involved in signal transduction or transcription factors were absent (ERK1/2 being an exception). Moreover, transport and hypoxic response (HSF1) pathways were overexpressed only in L428 cells. Interestingly, HSF1 regulates the heat shock response pathway, and HSF proteins were identified as the main group of proteins altered in L428 cells.

### 2.4. Overview of Proteins Modulated by Celastrol in KM-H2 and L428 Cells

Celastrol directly or indirectly modulated the expression of proteins with key cellular roles in cancer, with contrasting patterns of protein expression in the studied cell lines, characterized by upregulated proteins predominating in KM-H2 cells and downregulates proteins predominating in L428 cells (Table 1 and Tables S1–S4).

A set of proteins was found repeated in the top 10 most relevant pathways in KM-H2 cells. Four of these proteins were downregulated (RAS, ERK1, ERK2, and p90RSK1), while two were upregulated (PI3K cat class IA and cytochrome c) in celastrol-treated KM-H2 cells. RAS is an oncogenic protein upstream of the MAPK/ERK and PI3K/Akt signaling pathways [19]. ERK1/2 and P13K1A proteins are essential components of these signaling cascades and play critical roles in cellular proliferation, prevention of apoptosis, cell cycle arrest, and induction of drug resistance in human cancer [19]. p90RSK1 is a serine/threonine kinase and a downstream target of the MAPK/ERK pathway [20]. Finally, cytochrome c is a small hemeprotein that functions as a central component of the electron transport chain in mitochondria and is also involved in the initiation of apoptosis [21]. HSP90 inhibitors (e.g., geldanamycin) were found to destabilize and degrade virtually all HSP90 co-chaperones and their client proteins in cancer cells, including Refs. [22,23,24]. Celastrol, on the other hand, targets the HSP90/Cdc37complex, disrupting the interaction which is necessary for RAS stabilization [10,25]. Therefore, it is possible that HSP90 inhibition by celastrol leads to depletion of RAS, resulting in MAPK/ERK pathway inhibition in KM-H2 cells. Notably, it has been described that the MAPK/ERK pathway is constitutively active in H-RS cells [26]. Thus, our proteomic results suggest that celastrol may sensitize KM-H2 cells to apoptosis via the Raf/MEK/ERK pathway.

Interestingly, ERK1, ERK2, and p90RSK1 were also downregulated in L428 cells, indicating that targeting of the MAPK/ERK pathway may be one of the central celastrol mechanisms in H-RS cells. We also identified HSP60, an important stress-induced molecular chaperone [27], downregulated in celastrol-treated L428 cells, whereas AHA1 (activator of HSP90 ATPase-1), HSP70, HSP105, BAG2 (BCL2 Associated Athanogene 2), HSP27, and proteins involved in cytoskeleton organization and cell adhesion (α1-actin, α- and β-tubulin, Actin β, Myosin, and Contactin 1) were found upregulated. AHA1 is an HSP90 co-chaperone that has been suggested to act as a general stimulator of HSP90 function [28]. HSP105 and HSP70 are molecular chaperones of the HSP70 family that have been described as overexpressed in a variety of human tumors [29,30]. The co-chaperone BAG2 modulates the activity of HSP70/HSC70 by promoting substrate release; most previous studies on BAG2 emphasize its important role in cancer cell metastasis [31,32]. Finally, HSP27 is a heat-shock protein often associated with drug resistance, metastasis, and poor prognosis in human cancer. HSP27 acts as an anti-apoptotic agent in response to chemicals by inhibiting key effectors of the apoptotic machinery at the pre- (interacting with BCL2 associated X, apoptosis regulator; BAX and cytochrome c) and post-mitochondrial (preventing the apoptosome formation) level [30]. HSP27 also has the ability to regulate actin cytoskeletal dynamics during stress conditions [33], which explains, at least in part, the abundance of proteins involved in cytoskeleton organization that we detected in our proteomic analyses. These data collectively confirm that celastrol modulates chaperones and co-chaperones involved in oxidative and chemical stress and place those proteins in the center of the response and resistance to celastrol in cHL. This led us to hypothesize that, in this system, the upregulation of HSP27 would also have a cytoprotective role against cell death induction by celastrol.

### 2.5. Validation of the Proteomic Results 

**Figure 2:**
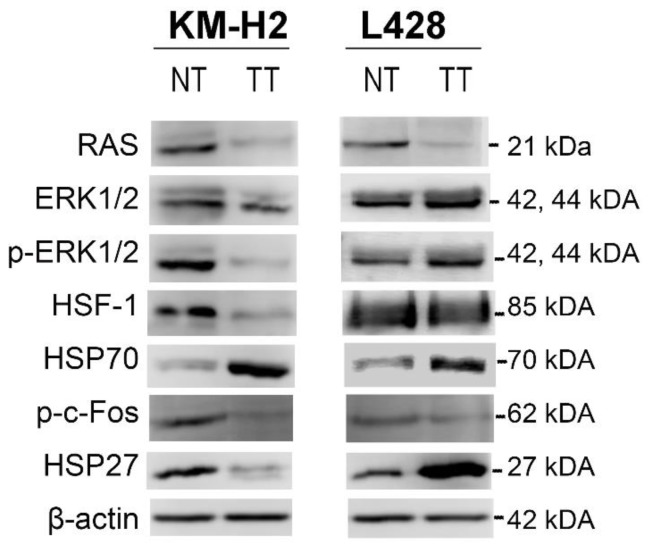
Western blot analyses of differentially expressed proteins found in the proteomic study. KM-H2 and L428 cells were treated with 1 µM of celastrol for 24 h for the validation of potential markers derived from the quantitative MS data. Samples (30 μg) were separated by SDS-PAGE (Sodium dodecyl sulfate polyAcrylamide gel electrophoresis) and probed with specific antibodies, as indicated. NT: non-treatment. TT: treatment.

To validate the mass-spectrometry (MS)-based proteomic results and our hypothesis, we performed western blot analysis of five proteins selected on the basis of our in-silico analyses: RAS, ERK1/2, p-ERK1/2, HSP70, and HSP27 (Figure 2). We also evaluated HSF-1 because it is the major transcription factor for HSPs proteins and c-Fos, a downstream ERK kinase target (Figure 2). In KM-H2 cells, treatment with 1.0 µM celastrol for 24 h led to a marked decrease in the protein levels of RAS and p-ERK1/2 and to an increase of HSP70, thus corroborating the proteomic results. The treatment also resulted in a decrease of HSF-1 protein levels. By using an antibody able to detect the phosphorylated (activated) protein, decreased levels of phospho-c-Fos (p-c-Fos) were observed after celastrol treatment. Together, our results indicate that celastrol can promote the apoptosis in KM-H2 cells by down-modulating the MAPK/ERK pathway, providing a proof of principle that the inhibition of the MAPK/ERK pathway may have therapeutic value in cHL.

In L428 cells, no differences were found in ERK1/2 or p-ERK1/2 (phospho-ERK1/2) protein levels in western blot assays between TT and NT conditions, unlike the results of the MS proteomics. This divergence is likely due to differences in sensitivity between proteomic (scale in femtomole) and western blot analyses (micrograms).

No variation in HSF-1 or p-c-Fos levels between TT and NT was found in L428 cells. Nevertheless, celastrol treatment led to an increase in HSP70 protein levels. Finally, although the basal levels of HSP27 were similar in KM-H2 and L428 cells (Figure 2), expression levels of HSP27 decreased in KM-H2 cells after celastrol exposure, whereas a prominent increase was seen in L428 cells. This biological event was accompanied by a marked, eightfold upregulation of *HSBP1* (HSP27) mRNA in the resistant L428 cell line (Figure S2). This suggests that resistance to celastrol may emerge in part as a compensatory mechanism involving the activation of HSP27.

## 3. Discussion

This study provides the first evidence of the potential role of celastrol, a HSP90 inhibitor [10,25], in regulating the growth and survival of H-RS cells. cHL is a remarkably heterogeneous disease [1] with scarce experimental models. At present, 4–5 H-RS-derived cell lines are available, and we describe disparate anti-proliferative and anti-apoptotic effects of the compound in two of those Hodgkin cell lines.

In cHL, HSP90 appears to be more than just a regular chaperone modulated in response to cellular stress [17]. Robust experimental evidences indicate that induction of HSP90 is mediated by CD30 and that HSP90 is a central hub for signaling integration in cHL cells [17]. HSP90 is abundantly expressed in cHL-derived cell lines and in >95% of primary Hodgkin lymphoma tumors [34]. Importantly, the HSP90 inhibitor geldanamycin and its derivative 17-AAG (17-allylamino-17-demethoxygeldanamycin) are able to induce cell death and to synergize with the effects of chemotherapy drugs in cHL cell lines [35,36]. Therefore, the results obtained in this work together with those of recent publications outline HSP90 as a potential therapeutic target in cHL.

In this work, we describe two different responses exhibited by cHL cell lines to the same compound. In the first place, in KM-H2 cell line celastrol induced growth inhibition, G0/G1 phase blockage, and finally, apoptosis. Induction of cytotoxicity is a well-known effect of celastrol in many cell lines derived from different hematological cancers, such as human lymphoblastoid cells, multiple myeloma, leukemia, as well as prostate, renal, and breast cancers [13,14,37,38]. However, in lung and hepatocellular carcinoma cell lines, celastrol is able to induce apoptosis and necrosis via ROS (reactive oxygen species) accumulation and G2-M phase blockage [39].

In the second place, in L428 cells, celastrol treatment did not result in significant cell growth inhibition, cell cycle arrest, or caspase-3/7 activation, even at high doses (5 µM).The observed apoptotic response in L428 (~30% at 5 µM) occurred mainly in a late phase and may be attributed to the high dosage [40].The non-responsiveness of L428 cells could be ascribed to TP53 mutation status; however, no strong evidence of p53 involvement came from our proteomic analysis. Besides, apoptosis induction by the HSP90 inhibitor geldanamycinin in p53mt HL cell lines has shown to be p53-independent [36]. Thus, the mechanisms responsible for resistance to celastrol are likely multifactorial and diverse. As far as we know, celastrol resistance is not a common phenomenon, and only a few cell lines, derived from glioblastoma, have been described to be celastrol-resistant [41]. Having an in vitro model of resistance is important to uncover the pathways and molecules involved in resistance to celastrol and, in a general manner, to HSP90 inhibitors.

In order to disclose a differential regulation of cellular processes, protein networks, and potential toxicological pathways modulated by celastrol in our experimental system, we opted for a high-throughput quantitative and label-free proteomic approach. We were able to show that celastrol perturbed multiple signaling pathways, involving mainly the MAPK kinase pathway, metabolism, dysregulation of protein folding, proteolysis, protein trafficking, and cytoskeleton organization. However, the major disclosed effect was to modulate protein homeostasis and stress response pathways. Hansen et al. conducted a study on celastrol effects in human lymphoblastoid cells and found that celastrol substantially modified the proteome composition, promoting proteotoxic stress [14]. As in our study, the authors identified that the altered proteins play key roles in cytoprotection, with a prominent group involved in folding and processing of endoplasmic reticulum proteins. Taken together, our proteomic findings are consistent with previous reports in which celastrol mainly promoted proteotoxic stress by disturbing protein homeostasis and heat shock response to stress [14,42].

In our system, the response to celastrol was associated with downregulated expression of RAS, ERK1/2, and c-Fos, suggesting that the compound suppressed the MAPK/ERK pathway. This was already reported as a mechanism to promote H-RS cell survival [26]. Moreover, the pharmacological inhibition of HSP90 in cHL cell lines has been shown to involve MAPK/ERK pathway downregulation [35]. In our system, celastrol treatment of KM-H2 cells lead to downregulation of HSF-1, a protein that is strongly expressed in H-RS cells [34] and plays a pivotal role in preserving protein stability [43]. This result highlights the proteostatic disturbance mediated by celastrol in H-RS cells.

Celastrol treatment of L428 cells resulted in increased expression of chaperones and co-chaperones. Among these, we focused on HSP27 because of its implication in apoptosis inhibition and drug resistance in tumor cells [44,45,46]. A correlation was observed between HSP27 expression and the response or resistance to celastrol in cHL cell lines, with L428 cells displaying a strong increase in HSP27 levels and KM-H2 cells showing a prominent decrease at protein and mRNA levels. Our results point to a mechanism of stress tolerance and celastrol resistance involving HSP27 in L428 cells. Evidence of this would be the upregulation of proteins involved in cytoskeleton organization (α1-actin, α- and β-tubulin, ACTB, myosin, and CNTN1), having in mind that the cytoprotective effect of HSP27 has been attributed to its capacity to bind actin and stabilize actin polymerization [47]. HSP27 also interferes with the cytochrome c-dependent mitochondrial apoptotic pathway, preventing the formation of the apoptosome and blocking the subsequent apoptosis cascade [45]. Nevertheless, we are aware that functional studies are required to demonstrate the role of HSP27 expression in celastrol’s resistance in H-RS cells.

Most HSP90 inhibitors lead to the overexpression of stress proteins like HSP27, which confer tumor cell survival and treatment resistance [48]. In fact, several HSP90 inhibitors with potent antitumor activity in preclinical models failed the clinical trials [48], mostly because of the induction of a stress response involving HSF-1, with subsequent increased levels of HSP70, HSP27, and clusterin. With respect to this, naturally occurring mechanism of cancer drug resistance may turn to be treatment targets, and strategies that address drug resistance could have significant therapeutic value. In fact, a recent study, with impressive results, showed that incorporation of 19S proteasome subunits allowed cancer cells to withstand the exposure to proteasome inhibitors; subsequently, when the regulatory subunits were reduced, the proteasome shifted to a state that protected the cells [49].

In the setting of HSP-targeted therapies, the hypothesis that the induction of HSP27, in reason of HSP90 inhibition, is a mechanism to bypass treatment-induced apoptosis has already been tested in prostate cancer [48]. In a model of castrate-resistant prostate cancer, a targeted strategy using OGX-427 (Apatorsen; a 2′-methoxyethyl-modified antisense oligonucleotide that inhibits Hsp27 expression) in combination with HSP90 inhibitors was able to stop tumor growth in vitro and prolong survival in vivo [48].

In cHL, HSPs are overexpressed [34,50], with >95% of cases displaying strong expression of HSF-1, HSP60, HSP90, HSP110/105, and HSP10, whereas HSP27 and HSP70 are expressed by a smaller fraction of cases (55.4% and 78.6%, respectively) [34]. More importantly, longer disease-free survival has been associated with a HSP27-negative status [34]. Although we have not evaluated HSPs expression in our cHL series, our findings in conjunction with those described above point to cHL as a possible candidate for therapies targeting the stress response.

## 4. Materials and Methods

### 4.1. Reagents and Drugs

All reagents and solvents used in this study are described in the Supplementary Files S1. Celastrol was dissolved at 50 mM in DMSO and stored at −20 °C to be used within three months after preparation. The stored solution was further diluted with RPMI 1640 (Roswell Park Memorial Institute 1640) medium to a work concentration.

### 4.2. Cell Culture and Treatments

Hodking Lymphoma (HL)-derived cell lines were kindly provided by Volker Diehl (University of Cologne): KM-H2 (DSMZ ACC 8) and L428 (DSMZ ACC 197) cell lines were established from the pleural effusion of a mixed cellularity and a nodular sclerosis cHL patient, respectively. After arrival to the laboratory, the phenotype and genotype of these cell lines were authenticated following current guidelines [51]. Mycoplasma infection was checked on a weekly basis by multiplex PCR assays, and cells were mycoplasma-free. Of note, KM-H2 is caspase-3 deficient and L428 carries an exon-4 TP53 in-frame deletion. Cells were cultured in RPMI-1640 and 10% heat-inactivated fetal bovine serum (FBS) in a humidified incubator containing 5% CO_2_ at 37 °C. Exponentially growing cells were used for the experiments. The cells were seeded in 96-well, 24-well culture plates, or 100 nm culture dishes at a density of 5 × 10^5^·mL^−1^ followed by exposure to the indicated doses of celastrol. The culture medium with DMSO (vehicle) served as a control; the final concentrations of DMSO never exceeded 0.1%. Each experiment was repeated at least three times.

### 4.3. Cell Viability Determined by WST-1

KM-H2 and L428 cells were seeded at 10^5^ cells/well in 96-well plates, and the cells were treated with celastrol (0.1, 0.25, 0.5, 1.0, 2.5, and 5.0 μM). After 12, 24, 36, or 48 h, the viable cells were detected by use of WST-1 (4-[3-(4-iodophenyl)-2-(4-nitrophenyl)-2*H*-5-tetrazolio]-1,3-benzene disulfonate sodium salt). The absorbance was measured at 450 nm versus 650 nm. Cell viability was expressed as a percentage with respect to untreated cells. 

### 4.4. Analysis of Caspase-3 and -7 Activities

For apoptosis induction, the cells were seeded 24 h before treatment in 96-well microplates. Subsequently, celastrol (0.5, 1.0, 2.5, and 5.0 μM) and DMSO were added to the cells for further 24 h. One hour before the end of drug exposure, Caspase-3/7-Glo reagent was added to the cells in a 1:2 dilution, and the cells were incubated for one hour at room temperature in the dark. The luminescence was measured at 700 nm. The background signal (medium and Caspase-3/7-Glo reagent only) was subtracted, and the data were normalized to DMSO-treated cells. Caspase activity was expressed as a ratio comparing the luminescence results of the treated cells to those of the control.

### 4.5. Flow Cytometry Analysis

Cell cycle analysis and apoptosis detection were performed after treating KM-H2 and L428 cells with celastrol at the described doses for 24 h. Cell cycle analysis was carried out by propidium iodide (PI) staining. Briefly, 2 × 10^5^ cells were fixed with ethanol 70% and incubated in 20 µg/mL PI and 0.2 mg/mL RNAse A for 30 min at room temperature. Apoptosis was detected by Annexin/PI assays. After being washed twice with cold PBS, the cells were incubated with 100 µL of binding buffer containing 5 µL Annexin V-FITC (recombinant annexin V conjugated to green-fluorescent FITC dye) and 10 µL PI (20 µg/mL). The samples were analyzed in a FACSCalibur Flow Cytometer (Becton Dickinson, Franklin Lakes, NJ, USA) collecting 10,000 events per analysis. DNA histograms and Annexin/PI dotplots were analyzed in CELLQuest software (FACSCalibur, version 5.1, Becton Dickinson, Franklin Lakes, NJ, USA).

### 4.6. Proteomic Studies

A total of 1 × 10^7^ cells from each cell line was cultured with or without celastrol, and protein extracts were obtained from homogenization with 100 μL of cold lysis buffer containing 50 mMTris-HCl (pH 7.5), 5 mM EDTA, 10 mM EGTA (ethylene glycol tetraacetic acid), 50 mM NaF, 20 mM KCl, and 250 mM NaCl supplemented with 1 μL of protease inhibitor cocktail. After 1 h at 4 °C, the suspension was frozen and thawed twice in liquid nitrogen. The total extracts were centrifuged at 12,000× *g* for 30 min, and the supernatants were collected and stored at −80 °C until further processing. Protein concentration was measured by Bradford assay, and the samples were concentrated 39× using a 3 kDa ultrafiltration device (Millipore, Billerica, MA, USA) with 50 mM NH_4_HCO_3_. In total, 200 μg of protein was used for tryptic digestion, as previously described [52].

Qualitative and quantitative nano-UPLC tandem nano-ESIHDMSE experiments were conducted with a nanoACQUITY UPLC system (Waters, Milford, MA, USA), as previously reported [52]. Briefly, a 180 μm × 23 mm strong cation exchange (SCX) column (Waters) packed with a 5 μm PolySULFOETHYL Aspartamide (PolyLC, Columbia, MD, USA) was used as the first dimension. Nine salt gradient fractions were used to elute the samples from the SCX column, followed by a reverse-phase (RP) gradient. After all of the peptides had been captured, the trap column was placed online with another RP analytical column (100 µm × 100 mm, 1.8 µm C18, nanoACQUITY UPLC HSS T3, Waters, Milford, MA, USA), and an RP gradient of 5–40% acetonitrile (ACN; containing 0.1% *v*/*v* formic acid) in 58 min was used as the second dimension, with a flow rate of 600 nL·min^−1^.

Analyses were performed using nano-electrospray ionization in positive ion mode, nano-ESI (+), with a NanoLockSpray ionization source (Waters, Milford, MA, USA). Multiplexed data-independent (DIA) scanning with added specificity and selectivity of a nonlinear “T-wave” ion mobility (HDMSE; Waters, Milford, MA, USA) experiments was performed with a Synapt HDMS mass spectrometer (Waters, Milford, MA, USA) [52]. Full-scan orthogonal acceleration time-of-flight (oaTOF) MSE data were acquired from *m*/*z* 50 to 2000.

Database searching and protein quantification were performed as previously described [52]. Briefly, Exact Mass Retention Time (ERMT) output tables generated for each condition (with or without celastrol) were filtered by considering a well-defined solitary peak without background noise (*OK* = 2) and differential expression profile (*p* = 1 up-regulated and *p* = 0 for downregulated). Additionally, a fold change higher than 50% (with or without celastrol <0.66 or >1.5) was considered to be indicative of significantly altered levels of expression. After filtering, protein data and associated expression profiles were used as input for the curated pathway database Metacore™ software (GeneGO Inc., Saint Joseph, MI, USA).

### 4.7. Western Blot Analysis

Cells were treated with celastrol (1 µM) or vehicle for 24 h, washed with PBS, and then lysed with lysis buffer (TrisHCl 50 mM pH 8.0, KCl 50 mM, EDTA 10 mM, NP40 1% supplemented with protease inhibitor cocktail, pH 7.4) for 30 min on ice. Thirty micrograms of each cell lysates (untreated and treated) was run on 15% sodium dodecyl sulfate-polyacrylamide gels (SDS-PAGE), transferred to nitrocellulose membranes (Roche, Mannheim, Germany), and probed with the indicated antibodies (Supplementary File S1). Detection was accomplished using the corresponding horseradish peroxidase (HRP)-conjugated secondary antibodies followed by development with ECL Plus Western Blotting Reagents (formerly Amersham Biosciences, Uppsala, Sweden).

### 4.8. qPCR Analysis

Total RNA was extracted (RNA Miniprep Systems, Promega Corporation, Madison, WI, USA), and 0.5 µg was used to synthesize first-strand cDNA with the High-Capacity cDNA Archive kit (Applied Biosystems by Life Technologies, Austin, TX, USA). The expression of *HSBP1* (HSP27) mRNA was detected via qPCR by use of SYBR™ Green PCR Master Mix (Applied Biosystems™) in an ViiA™ 7 Real-Time PCR System (Thermo Fisher Scientific, Carlsbad, CA, USA), using specific primers (forward 5′-CCCTGGATGTCAACCACTTC-3′ and reverse 5′-GATGTAGCCATGCTCGTCCT-3′). *GUSB* was selected as a reference gene on the basis of its low variability and stability determined with the Normfinder algorithm (forward 5′-CCTGTGACCTTTGTGAGCAA-3′ and reverse 5′-AACAGATCACATCCACATACGG-3′). The relative mRNA levels were calculated in fold changes as 2^−Δ*C*q^, after normalization with *GUSB* expression.

### 4.9. Statistical Analyses

The quantitative data were shown as mean ± SD from at least three different experiments. The IC50 values were calculated using nonlinear regression (curve fit), and the statistical comparison among groups was performed by ANOVA analysis followed by Bonferroni post-tests. The statistical significance of the differences between experimental groups was determined with the paired two-tailed Student’s *t* test. The statistical significance was set at *p* < 0.05. The analyses were performed by use of GraphPad Prism 5.0 software (GraphPad Software, Inc., CA, USA).

## 5. Conclusions

Our results provide in vitro evidence of different sensitivities to celastrol in cHL cells. The antitumor effects of celastrol proceed via the suppression of the MAPK/ERK pathway. Resistance to celastrol has rarely been described and, in cHL, it may be mediated by the upregulation of the small heath-shock protein HSP27. The effects of HSP90 inhibitors as antitumor compounds for cHL and whether the disparate responses observed in vitro have clinical correlates deserve further research. 

## Figures and Tables

**Table 1:** Representative pathways modulated by celastrol in KM-H2 and L428 cell lines and differentially expressed proteins present in these major pathways.

Pathway Name *	FDR	N#	Identified Proteins
**KM-H2 cell line**			
Development_Ligand-independent activation of ESR1 and ESR2	2.5 × 10^−5^	8/44	p300, ERK1/2, ERK1 (MAPK3), ERK2 (MAPK1), PI3KIA, PI3KIA t class IA (p110-alpha), p90RSK1, CBP
NETosis in SLE	3.9 × 10^−4^	6/31	ERK1/2, Histone H3, Histone H2, Histone H2A, Histone H1.2, Histone H1
Cell cycle_Role of Nek in cell cycle regulation	3.9 × 10^−4^	6/32	Histone H3, PI3K cat class IA, Tubulin, Tubulin beta, Histone H1, Tubulin alpha
Cytoskeleton remodeling_Neurofilaments	1.6 × 10^−3^	5/25	Vimentin, Tubulin (in microtubules), Tubulin beta, Desmuslin, Tubulin alpha
Signal transduction_Additional pathways of NF-kB activation	2.9 × 10^−3^	5/30	p300, ERK1/2, Histone H3, p90RSK1, CBP
Development_IGF-1 signaling	2.9 × 10^−3^	6/50	ERK1/2, ERK1 (MAPK3), ERK2 (MAPK1), PI3K cat class IA, NF-kB, CDC42
Sorafenib-induced inhibition of cell proliferation and angiogenesis in HCC	2.9 × 10^−3^	4/16	VEGFR-1, ERK1/2, ERK1 (MAPK3), ERK2 (MAPK1)
Cell cycle_Start of DNA replication in early S phase	2.9 × 10^−3^	5/32	RPA3, MCM3, Histone H1, MCM5, MCM2
Signal transduction_Activin A signaling regulation	2.9 × 10^−3^	5/33	p300, Histone H3, Evi-1, Histone H2, CBP
Development_S1P1 receptor signaling via beta-arrestin	2.9 × 10^−3^	5/34	ERK1/2, ERK1 (MAPK3), ERK2 (MAPK1), PI3K cat class IA (p110-alpha), p90Rsk
**L428 cell line**			
Regulation of degradation of deltaF508-CFTR in CF	3.5 × 10^−5^	8/39	HSP70, HSP105, HSP27, SUMO-2, E2I, Aha1, SAE1, BAG-2
NETosis in SLE	4.3 × 10^−4^	7/31	ERK1/2, Histone H3, Histone H2A, Histone H2, Histone H1, Histone H1.2, HMGB1
Transcription_Negative regulation of HIF1A function	4.9 × 10^−4^	8/66	HSP70, MCM7, PSMA7, PRDX4, RUVBL2, MCM2, MCM5, PRDX2
Cell cycle_Start of DNA replication in early S phase	1.2 × 10^−3^	6/32	MCM4/6/7 complex, RPA3, MCM2, MCM4, Histone H1, MCM5
Development_Regulation of cytoskeleton proteins in oligodendrocyte differentiation and myelination	1.6 × 10^−3^	7/58	Tubulin alpha, Tubulin, Actin cytoskeletal, Tubulin beta, Dcc, MRLC, Cortactin
Cytoskeleton remodeling_Neurofilaments	2.5 × 10^−3^	5/25	Tubulin alpha, Tubulin, Actin cytoskeletal, Tubulin beta, Kinesin heavy chain
Immune response_Sublytic effects of membrane attack complex	3.1 × 10^−3^	7/68	RK1/2, GRP75, HSP27, Actin cytoskeletal, cPLA2, GRP78, eIF2S1
Development_Slit-Robo signaling	3.1 × 10^−3^	5/30	Tubulin, Actin cytoskeletal, Actin, ACTB, Cortactin
Transport_The role of AVP in regulation of Aquaporin 2 and renal water reabsorption	3.5 × 10^−3^	6/50	ERK1/2, Actin cytoskeletal, ACTB, MRLC2, MRLC, Annexin II
Cell cycle_Role of Nek in cell cycle regulation	3.5 × 10^−5^	5/32	Tubulin alpha, Tubulin, Histone H3, Tubulin beta, Histone H1

* Pathways listed in the table are those statistically most relevant identified by using the Metacore software (GeneGO, Encinitas, CA, USA). FDR: False discovery rate. N#: proteins data/total. ESR1 (estrogen receptor 1), ESR2 (Estrogen receptor 2), SLE (Systemic lupus erythematosus), NEK (NIMA related kinase), IGF1 (Insulin growth factor 1), HCC (Hepatocellular Carcinoma), S1P1 (Sphingosine-1-phosphate receptor 1), CF (Cystic fibrosis) and AVP (Arginine vasopressina).

## Supplementary Materials

Supplementary materials can be found at http://www.mdpi.com/1422-0067/19/3/836/s1.
