# Supplementary material for: Targeting Hodgkin and Reed–Sternberg Cells with an Inhibitor of Heat-Shock Protein 90: Molecular Pathways of Response and Potential Mechanisms of Resistance
Source: Int J Mol Sci. 2018 Mar 13;19(3):836. doi: 10.3390/ijms19030836 (PMC5877697; doi:10.3390/ijms19030836)
Supplement: Supplementary file 1 [file ijms-19-00836-s001.zip › Supplementary Figure S2.pdf]

International Journal of Molecular Sciences

Targeting Hodgkin and Reed-Sternberg cells with an inhibitor of heat-shock protein 90: Molecular pathways of response and potential mechanisms of resistance

Priscilla Segges[a], Stephany Corrêa[b], Bárbara Du Rocher[b,c], Gabriela Vera-Lozada[a], Flavia Krsticevic[a,d], Debora Arce[d,e], Cinthya Sternberg[f,g], Eliana Abdelhay[b], Rocio Hassan[a]

[a]Oncovirology Laboratory, Bone Marrow Transplantation Center (CEMO), Instituto Nacional de Câncer (INCA), Rio de Janeiro, Brazil; [b]Laboratório Célula-Tronco, CEMO, Instituto Nacional de Câncer (INCA), Rio de Janeiro, Brazil; [c]Laboratório de Pesquisa sobre o Timo, FIOCRUZ, Rio de Janeiro, Brazil; [d]CIFASIS - Centro Internacional Franco Argentino de Ciencias de la Información y de Sistemas, Rosario, Argentina; [e]IICAR-CONICET Instituto de Ciencias Agrarias de Rosario, Rosario, Argentina; [f]Laboratório de Pesquisa Translacional, Instituto Nacional de Câncer (INCA), Rio de Janeiro, Brazil; [g]current addres: Programa de Pós-Graduação em Anatomia Patológica, Faculdade de Medicina da Universidade Federal do Rio de Janeiro, Brazil.

CORRESPONDENCE TO: Priscilla Segges.

Instituto Nacional de Câncer, Praça Cruz Vermelha, n° 23, 6° andar, ala C, CEMO, Laboratório de Oncovirologia, CEP: 20230130, Rio de Janeiro, RJ, Brazil.

Telephone: 5521-3207-1874 Fax: 5521-3207-2121

Email: psegges@inca.gov.br

Supplementary Figure S2

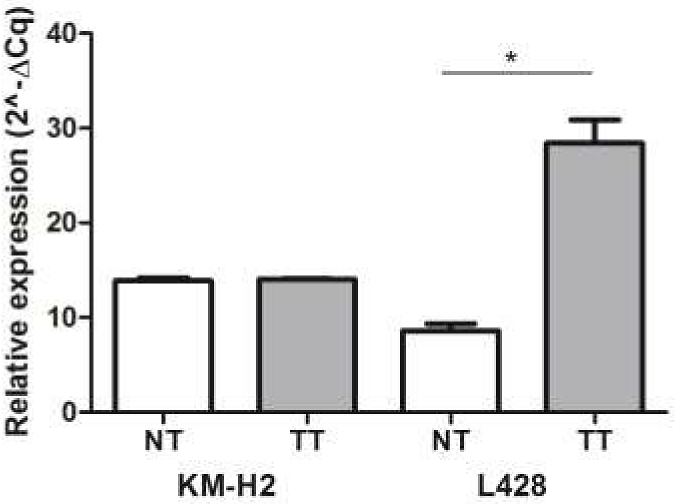

Figure S2: Analysis of *HSP27* mRNA expression. NT: untreated and TT: treated. Bars represent standard error. * $P < 0.001$ (Student t test).
