# Supplementary material for: Targeting Hodgkin and Reed–Sternberg Cells with an Inhibitor of Heat-Shock Protein 90: Molecular Pathways of Response and Potential Mechanisms of Resistance
Source: Int J Mol Sci. 2018 Mar 13;19(3):836. doi: 10.3390/ijms19030836 (PMC5877697; doi:10.3390/ijms19030836)
Supplement: Supplementary file 1 [file ijms-19-00836-s001.zip › Supplementary File S1.pdf]

International Journal of Molecular Sciences

Targeting Hodgkin and Reed-Sternberg cells with an inhibitor of heat-shock protein 90: Molecular pathways of response and potential mechanisms of resistance

Priscilla Segges[a], Stephany Corrêa[b], Bárbara Du Rocher[b,c], Gabriela Vera-Lozada[a], Flavia Krsticevic[a,d], Debora Arce[d,e], Cinthya Sternberg[f,g], Eliana Abdelhay[b], Rocio Hassan[a]

[a]Oncovirology Laboratory, Bone Marrow Transplantation Center (CEMO), Instituto Nacional de Câncer (INCA), Rio de Janeiro, Brazil; [b]Laboratório Célula-Tronco, CEMO, Instituto Nacional de Câncer (INCA), Rio de Janeiro, Brazil; [c]Laboratório de Pesquisa sobre o Timo, FIOCRUZ, Rio de Janeiro, Brazil; [d]CIFASIS - Centro Internacional Franco Argentino de Ciencias de la Información y de Sistemas, Rosario, Argentina; [e]IICAR-CONICET Instituto de Ciencias Agrarias de Rosario, Rosario, Argentina; [f]Laboratório de Pesquisa Translacional, Instituto Nacional de Câncer (INCA), Rio de Janeiro, Brazil; [g]current addres: Programa de Pós-Graduação em Anatomia Patológica, Faculdade de Medicina da Universidade Federal do Rio de Janeiro, Brazil.

CORRESPONDENCE TO: Priscilla Segges.

Instituto Nacional de Câncer, Praça Cruz Vermelha, n° 23, 6° andar, ala C, CEMO, Laboratório de Oncovirologia, CEP: 20230130, Rio de Janeiro, RJ, Brazil. Telephone: 5521-3207-1874 Fax: 5521-3207-2121 Email: psegges@inca.gov.br

Supplementary File 1

Reagents

All reagents and solvents employed were commercially available and used as recommended by the manufacturers. RPMI 1640, streptomycin/penicillin for cell culture, 0.4% trypan blue vital stain, propidium iodide (PI), RNase and dimethyl sulfoxide (DMSO) were purchased from Sigma-Aldrich Co. (St. Louis, MO). 4-[3-(4-iodophenyl)-2-(4-nitrophenyl)-2H-5-tetrazolio]-1,3-benzene disulfonate (WST-1), protease inhibitor cocktail and ECL Plus® for western blot were purchased from Roche (Mannheim, Germany). Fetal bovine serum (FBS) was obtained from Gibco Life Technologies (Grand Island, NY). Annexin V-FITC Apoptosis Detection Kit and Caspase-Glo® 3/7 Assay Kit and ReliaPrep™ were purchased from BD Biosciences (Franklin Lakes, NJ) and Promega Corporation (Madison WI), respectively.

Antibodies against phospo-p44/42 (p-ERK1/2, D13.14.4E), p44/42 (ERK1/2), RAS (27H5), HSP70, HSF-1, HSP27 (G31), and anti-rabbit or mouse IgG HRP-linked were purchased from Cell Signaling Technology, Inc (Bervely, MA). Anti-actin and phospo-c-Fos antibodies were obtained from Santa Cruz Biotechnology (Santa Cruz, Calif) and Abcam (Cambridge, MA), respectively.

Celastrol with purity greater than 98% was purchased from Cayman Chemical (Ann Arbor, MI, USA).
