# Supplementary material for: Targeting Hodgkin and Reed–Sternberg Cells with an Inhibitor of Heat-Shock Protein 90: Molecular Pathways of Response and Potential Mechanisms of Resistance
Source: Int J Mol Sci. 2018 Mar 13;19(3):836. doi: 10.3390/ijms19030836 (PMC5877697; doi:10.3390/ijms19030836)
Supplement: Supplementary file 1 [file ijms-19-00836-s001.zip › Supplementary Table S5.pdf]

International Journal of Molecular Sciences

Targeting Hodgkin and Reed-Sternberg cells with an inhibitor of heat-shock protein 90: Molecular pathways of response and potential mechanisms of resistance

Priscilla Segges[a], Stephany Corrêa[b], Bárbara Du Rocher[b,c], Gabriela Vera-Lozada[a], Flavia Krsticevic[a,d], Debora Arce[d,e], Cinthya Sternberg[f,g], Eliana Abdelhay[b], Rocio Hassan[a]

[a]Oncovirology Laboratory, Bone Marrow Transplantation Center (CEMO), Instituto Nacional de Câncer (INCA), Rio de Janeiro, Brazil; [b]Laboratório Célula-Tronco, CEMO, Instituto Nacional de Câncer (INCA), Rio de Janeiro, Brazil; [c]Laboratório de Pesquisa sobre o Timo, FIOCRUZ, Rio de Janeiro, Brazil; [d]CIFASIS - Centro Internacional Franco Argentino de Ciencias de la Información y de Sistemas, Rosario, Argentina; [e]IICAR-CONICET Instituto de Ciencias Agrarias de Rosario, Rosario, Argentina; [f]Laboratório de Pesquisa Translacional, Instituto Nacional de Câncer (INCA), Rio de Janeiro, Brazil; [g]current addres: Programa de Pós-Graduação em Anatomia Patológica, Faculdade de Medicina da Universidade Federal do Rio de Janeiro, Brazil.

CORRESPONDENCE TO: Priscilla Segges.

Instituto Nacional de Câncer, Praça Cruz Vermelha, n° 23, 6° andar, ala C, CEMO, Laboratório de Oncovirologia, CEP: 20230130, Rio de Janeiro, RJ, Brazil.

Telephone: 5521-3207-1874 Fax: 5521-3207-2121

Email: psegges@inca.gov.br

Supplementary File S7

Table S7 Representative Biological process modulated in KM-H2 and L428 cell lines.

#	Biological processes	FDR	N# proteins in total	N# proteins identified
	**KM-H2 cell line**			
1	posttranscriptional regulation of gene expression	5,542E-13	669	39
2	chromosome organization	9,420E-12	1344	53
3	organelle organization	9,420E-12	4189	103
4	regulation of organelle organization	1,119E-11	1713	60
5	regulation of cellular component organization	1,189E-11	3298	88
6	cellular protein metabolic process	1,189E-11	4577	108
7	Fc receptor signaling pathway	1,316E-11	361	27
8	cellular component organization or biogenesis	1,316E-11	7183	144
9	cell cycle	7,472E-11	1770	59
10	cellular metabolic process	7,518E-11	10816	186
	**L428 cell line**			
1	translational initiation	1,170E-24	173	32
2	SRP-dependent cotranslational protein targeting to membrane	1,079E-20	103	24
3	protein localization to endoplasmic reticulum	1,080E-20	148	27
4	viral gene expression	1,553E-20	136	26
5	nuclear-transcribed mRNA catabolic process, nonsense-mediated decay	1,553E-20	137	26
6	viral transcription	1,691E-20	124	25
7	cotranslational protein targeting to membrane	1,691E-20	110	24
8	protein targeting to ER	7,170E-20	117	24
9	establishment of protein localization to endoplasmic reticulum	1,841E-19	122	24
10	cellular component biogenesis	9,278E-19	3351	106

*Process listed in the table are those statistically most relevant using the Metacore Analysis. FDR: *False discovery rate.*
